# Functional connectivity of the superior temporal sulcus at term-equivalent age: effects of gestational age and sex

**DOI:** 10.1007/s00429-025-02979-5

**Published:** 2025-07-26

**Authors:** Charlotte Mancuso, Maxime Bacquet, Lucas Benjamin, François Leroy, Ghislaine Dehaene-Lambertz

**Affiliations:** 1 Cognitive Neuroimaging Unit, CNRS ERL 9003, INSERM U992, CEA, Université Paris-Saclay, Robert Debré Child Brain Institute, NeuroSpin Center, Gif/Yvette, France; 2 Ecole Normale Supérieure de Lyon, Université Claude Bernard Lyon I, Université de Lyon, Lyon Cedex 07, 69342 France

**Keywords:** Prematurity, Brain, Infant, Resting-state, Language, Speech, Voice, Sex, Noise

## Abstract

The superior temporal sulcus (STS) plays a central role in auditory and linguistic processing and undergoes rapid development during the last trimester of gestation. Yet, the extent to which its development is shaped by early sensory experience remains unclear. Premature birth offers a unique opportunity to address this question, as it exposes the brain to an extra-uterine auditory environment at a critical stage of network maturation. We analyzed resting-state fMRI data in 116 neonates (63 males), scanned at term-equivalent age but born at varying gestational age (24.3 to 41.7 weeks gestational age) using the developing Human Connectome Project (dHCP) database. Functional connectivity was computed in native space using regions of interest based on each infant’s sulcal anatomy to assess the respective contributions of STS subregions. Our analyses reveal a functional division between the inferior and superior banks of the STS, with the inferior bank showing stronger connectivity to distant parietal and frontal areas along the dorsal language pathway. The left posterior STS emerged as a functional hub, displaying broad inter-area connectivity. Longer gestations correlated with increased local connectivity, notably in the right temporal region, despite equal age at scan. Additionally, female neonates exhibited stronger connectivity from the left posterior STS compared to males. These findings highlight the early emergence of adult-like auditory-linguistic networks and their sensitivity to the *in-utero* environment. Further research is needed to investigate the consequences of these early differences and to determine which postnatal interventions might help compensate, if necessary.

## Introduction

Brain responses to sound, including speech discrimination, are observed as early as 30 weeks of gestational age (wGA) in preterm neonates as in fetuses (Baldoli et al. 2015; Draganova et al. 2005; Jardri et al. 2008; Mahmoudzadeh et al. 2013; Moser et al. 2021). At birth, full-term neonates recognize their mother’s voice (DeCasper and Fifer 1980) and their native language (Mehler et al. 1988) indicating that they have encoded salient features of their *in-utero* auditory environment. These early perceptual abilities suggest that, by the last trimester of gestation, the auditory and linguistic networks are sufficiently developed to already support complex sound processing.

In older infants and adults, sound processing relies on parallel and hierarchical processing pathways extending from Heschl’s gyrus to the superior temporal gyrus (Rauschecker and Scott 2009). The superior temporal sulcus (STS) occupies a crucial position in this architecture, being the first region to show selective activation to the linguistic- and voice-values of speech compared to other auditory stimuli (Belin et al. 2000; Blasi et al. 2011; Dehaene-Lambertz et al. 2005; DeWitt and Rauschecker 2012). Along its anterior–posterior axis, speech is processed via two major pathways: a dorsal pathway, connected to frontal language areas via the arcuate fasciculus—a strongly left-lateralized tract in both adults and infants (Dubois et al. 2009; Thiebaut de Schotten et al. 2012)—supports verbal working memory and articulatory-phonetic representations, while a ventral pathway comprising the uncinate fasciculus and inferior fronto-occipital fasciculus, has been more closely associated with semantic processing and speech comprehension. To what extent early sensory experience shapes this architecture remains unclear. Although intrinsic maturational programs clearly drive certain aspects of the developing language network—such as the sensitive period for acquiring the phonology of the native language (Peña et al. 2012; Peña et al. 2010)—early auditory experience may also contribute to fine-tuning emerging connectivity.

Premature birth offers a natural model to explore this question, as it exposes the immature brain to an auditory environment that differs markedly from the intrauterine one. Unlike the filtered, rhythmic soundscape of the womb, the NICU environment introduces constant mechanical noise, abrupt alarms, and reduced exposure to maternal speech. If the infant is placed in an incubator, speech exposure may drop to ~ 32 min per day, compared to ~ 2.6 h in utero, and voice frequencies are also more attenuated (Monson et al. 2020, 2023). These differences might impact the maturation of auditory and language networks, particularly within the STS, which integrates inputs from both sensory and higher-order systems. Comparing infants scanned at term-equivalent age but born at varying gestational ages provides a unique opportunity to assess the potential impact of early auditory experience on the functional development of these networks.

Understanding the early development of these networks is all the more important given that children born prematurely are at greater risk for language disorders than those born at term (Guarini et al. 2009). Widespread alterations in functional connectivity (Eyre et al. 2021) as subtle anatomical disorganization in the superior temporal region and its connections have been reported in preterm infants at term-equivalent age. These alterations may contribute to later language difficulties (Aeby et al. 2013; Bartha-Doering et al. 2023; Kline et al. 2020; Salvan et al. 2017). These findings underscore the need for deeper investigation into the early functional organization of the auditory-linguistic network.

Therefore, we took advantage of the rich resources of the Developing Human Connectome Project (dHCP; http://www.developingconnectome.org) to explore the functional connectivity of the STS. This study was primarily exploratory with two main objectives: first, to characterize resting-state STS connectivity at term-equivalent age, with particular attention to the distinct contributions of the STS subregions; and second, to evaluate how early sensory experience may shape these emerging networks. We also considered the effect of biological sex, as male individuals—who are generally reported to have more asymmetric brains, a higher risk of cognitive impairment, and slower development of communication skills compared to females (Etchell et al. 2018; Fenske et al. 2023; Hirnstein et al. 2019; Leroy et al. 2015; Williams et al. 2021a)—may also exhibit distinct connectivity patterns and developmental trajectories.

A pioneering study suggested that resting-state connectivity in term neonates would initially be restricted to contralateral regions within the language network (Perani et al. 2011), in contrast to several activation studies that reported engagement of a broader network, including frontal regions when neonates listened to speech and music (Mahmoudzadeh et al. 2013; Perani et al. 2010, 2011). This discrepancy may reflect either genuinely weak early fronto-temporal coupling, or limited sensitivity of this early study. Recent research has emphasized that the detection of long-range anterior-posterior connectivity in neonates requires long, artifact-free recordings, due to the high sensitivity of these measurements to head pitch motion, frequent at this age (Sylvester et al. 2023). In this context, the dHCP database provides an ideal resource: its large sample size and optimized acquisition protocols for neonates significantly increase the sensitivity and reliability of resting-state connectivity analyses during this critical developmental period.

**Fig. 1:**
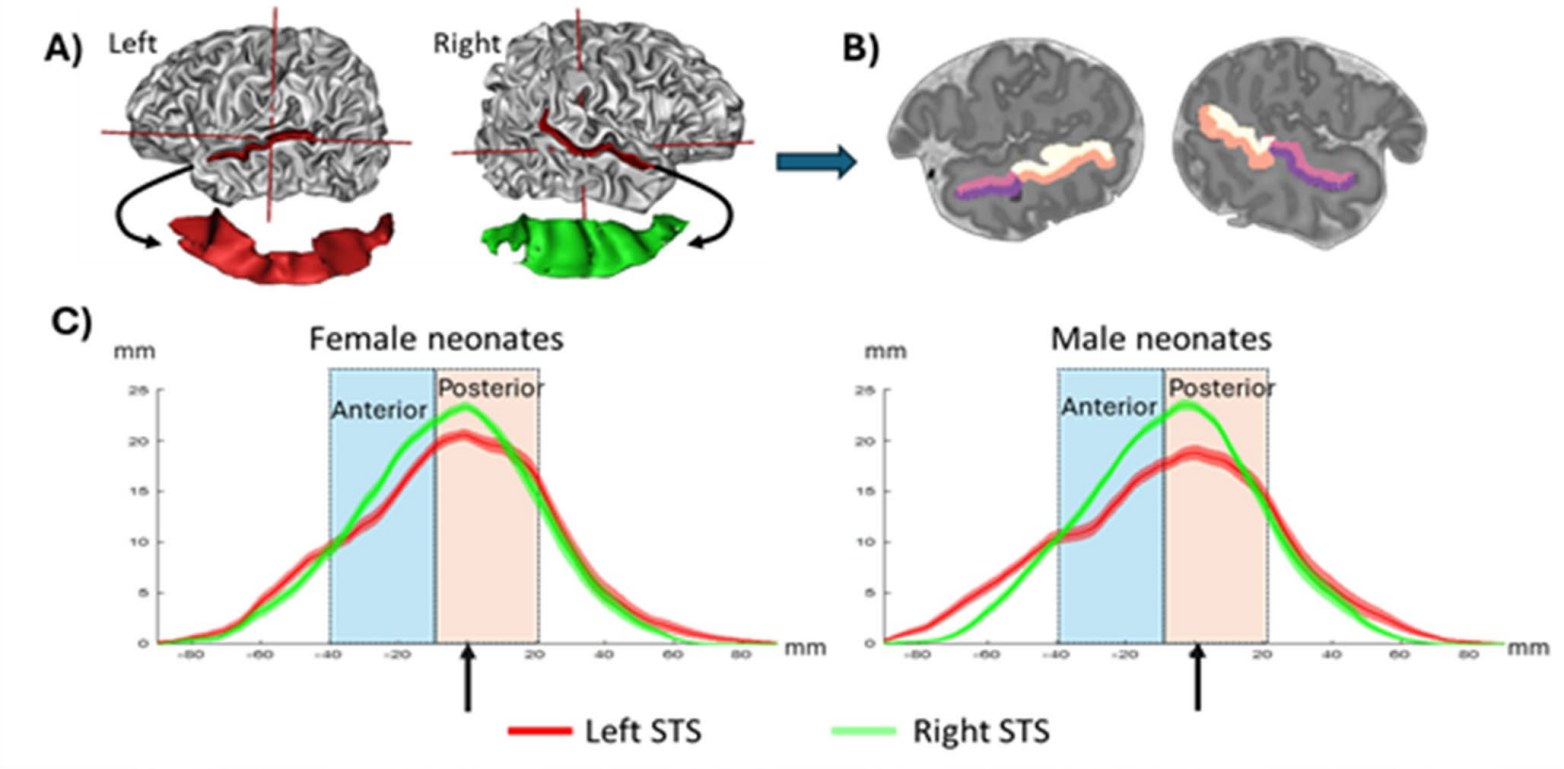
**A** Identification of the STS in an individual full-term neonate, shown on her 3D mesh (top row) and in isolation (bottom row). **B** Division of her STS into four regions of interest (ROIs), shown on a sagittal view. The inferior and superior ROIs correspond to the two banks of the STS within the STAP segment [− 40, + 20], which is further subdivided into anterior [− 40, − 10] and posterior [− 10, 20] ROIs. The STAP is the region of asymmetric depth between the right and left STS (Leroy et al. 2015). **C** Mean STS depth profile in male and female infants scanned at term-equivalent age. The shadow surrounding the line corresponds to the standard error of the mean. The coronal slice at the posterior tip of the *planum temporale* serves as the origin (arrow) for the anterior-posterior coordinates. The black dotted contours indicate the anterior and posterior boundaries of the ROIs

To further enhance sensitivity and anatomical precision, we reconstructed the STS shape in each infant’s native space and subdivided the individual masks into four ROIs (Fig. 1) aligned with the two principal organizational axes of the STS, to better capture its connectivity structure: (1) a superior/inferior division, chosen to reflect general auditory processing in the superior temporal gyrus versus more specialized processing in the inferior bank of the STS (Dehaene-Lambertz et al. 2002; DeWitt and Rauschecker 2012; Yeterian and Pandya 1991) and (2) an anterior/posterior division motivated both by the distinction between dorsal and ventral processing pathways and by the emergence during this period of a hemispheric asymmetry in STS depth, which may reflect the early differentiation of these dual anatomical routes (Bartha-Doering et al. 2023; Bonte et al. 2013; Leroy et al. 2015; Glasel et al. 2011). We also tested for left–right differences in STS connectivity, given the classically reported hemispheric asymmetries in language and voice processing (Belin et al. 2000; Pallier et al. 2011).

## Materials and methods

### Participants

All of the data used for the analysis were retrieved from a public database: “the developing Human Connectome Project dataset (dHCP) (release 2)” (www.developingconnectome.org). In this database, participants are preterm and full-term neonates, imaged at the Evelina Newborn Imaging Centre, St Thomas’ Hospital, London, UK. The document accompanying the release states that: “The study was approved by the UK Health Research Authority (Research Ethics Committee reference number: 14/LO/1169) and written parental consent was obtained in every case for imaging and data release”.

Release 2 of the dHCP database includes 558 MRI sessions from 505 subjects scanned between 24 and 45 weeks of gestational age (wGA) (see the release notes for full participant details: http://www.developingconnectome.org/release-notes). From this dataset, we included only infants scanned at term-equivalent age who had high-quality anatomical scans—based on the radiological quality control score provided with the dataset—and for whom functional data were available. We also ensured an even representation across the full range of gestational ages at birth. In the case of twins, one infant was randomly selected to avoid redundancy. Scans performed less than 48 h after birth were excluded to minimize the influence of early postnatal physiological factors (e.g., cortisol peak, hydration status) on brain segmentation. This selection process resulted in a final subset of 159 infants.

After performing additional quality controls on the functional data and excluding cases where the STS could not be reliably extracted from anatomical scans (see below), a total of 116 subjects (54.3% male neonates) were ultimately included in the analyses. The group comprised 31 infants born before 35 wGA (min = 24.3 wGA), 29 between 36 and 38 wGA, 31 between 38 and 40 wGA and 25 after 40 wGA (max = 41.7 wGA) (Table 1). No infants had any reported neurological impairments, and all were scanned at term-equivalent age (mean = 40.6 wGA [37–45.1]). For a post-hoc comparison of the effect of gestational age, we divided the group using 38 weeks of gestational age (wGA) as a cut-off, since early-term births (< 38 wGA) remain associated with an increase risk of complications, particularly in verbal and non-verbal communication, compared with later-term births (Hochstedler et al. 2021; Sengupta et al. 2013; Vohr 2013). We verified that at scan, term-equivalent age and cranial perimeter were not significantly different across the two groups (Table 1).

**Table 1:** Characteristics of the two subgroups divided at 38 wGA (mean and range)

	Infants born before 38wGA	Infants born after 38wGA	Cohen’s d	*P*-value^1^
N	60	56		
Gender (males)	30 (50%)	33 (59%)		0.3
Gestational age at birth (wGA)	33.2 [24.3–37.9]	39.7 [38.0–41.7]		
Birth weight (kg)	1.97 [0.54–4.10]	3.24 [2.16–4.70]	1.84	< 0.001
Chronological age at scan (weeks)	7.3 [0.1–17.1]	1.0 [0.1–4.3]	− 1.60	< 0.001
Postmenstrual age at scan	40.42 [37.00–45.15]	40.72 [38.14–44.86]	0.148	0.4
Cranial perimeter at scan (cm)	34.40 [26.1–39.0]	34.49 [30.5–38.0]	0.047*4*	0.8

^1^Welch two-samples *t* test, except for sex analysis which consisted in a Pearson’s Chi-squared test. Given the definition of the group based on gestational age at birth, the significant differences in chronological age at scan and birth weight were expected

### Data acquisition

MRI was performed on a 3 T Philips Achieva using a 32-channel phased array head-coil in a single session (63 min) comprising structural, functional and diffusions images during natural sleep (Hughes et al. 2017). We considered here the T2w anatomical image, because of its better signal to differentiate white and gray matter at such a young age relative to T1w, and the resting-state data images. The T2w images were acquired in axial and sagittal slices stack (0.8 × 0.8 × 1.6 mm with a slice overlap of 0.8 mm; TR/TE = 12000/156ms, SENSE factor = 2.11). Functional images were acquired using multiband 9x accelerated echo-planar imaging with high temporal resolution (TE/TR = 38/392ms, voxel size = 2.15 × 2.15 × 2.15 mm). The acquisition lasted for about 15 min (2300 volumes).

### Data processing

#### Anatomical processing and selection of the regions of interest (ROIs)

T2w images were acquired and preprocessed by the dHCP team (Cordero-Grande et al. 2016). Brain mesh and sulci shapes were extracted and automatically labeled using the BrainVisa software (Mangin et al. 2004; Perrot et al. 2011). M.B. visually inspected the results and corrected the labelling if necessary. The deepest point of the planum temporale was then identified. The origin of the STS coordinate system was determined following Leroy et al. (2015) and corresponded to the coronal plane displaying the deepest point of the *planum temporale*, a landmark shown to be relatively stable across individuals (Glasel et al. 2011). The same parametrization as in Leroy et al. (2015) was applied to define a geodesic coordinate system along the STS including antero-posterior and inner-outer axes. To use this method, a continuous volume is required. However, transverse gyri sometimes cut across the STS, disrupting its continuity (Ochiai et al. 2004). To address this, we created artificial junctions using the shortest available path.

We analyzed the functional connectivity of the STS in the native space. Our seeds were thus created individually for each neonate from their own STS using the individual sulci shapes created as explained above (Fig. 1). We focused our analyses on the superior temporal asymmetrical pit (STAP) region, defined in the human population by Leroy et al. (2015) as the segment spanning [− 40, + 20 mm] along the STS coordinate system (Fig. 1). This segment is significantly deeper in the right hemisphere than in the left and is present in 96% of humans but absent in non-human primates (Leroy et al. 2015). In adults, the STAP has been associated with specific activations in voice perception (Bodin et al. 2018) and speech comprehension (Ardellier 2018).

We dilated the STS shape with a factor of 3 mm to recover the cortical regions surrounding the sulcus, then binarized it to create masks. The masks were further divided into superior and inferior seeds, following the shape of the STS, as well as anterior [− 40, − 10 mm] and posterior [− 10, + 20 mm] regions. The anterior-posterior division was designed to account for the progressive age-related increase in the length of the asymmetric STS segment extending toward the temporal pole. The [−10 mm] boundary corresponds to the anterior boundary of the neonatal STAP, as determined using pilot data (Falières 2018). Specifically, the asymmetrical segment measured 30 mm in a preliminary analysis of 37 full-term neonates from the dHCP release 0 dataset, 45 mm in 3-month-old infants, and 55 mm in adults (Leroy et al. 2015). Consequently, the asymmetry is already visible in the posterior ROI [− 10, + 20 mm] from term-equivalent age onward, whereas it develops later in the anterior ROI [− 40, − 10 mm].

The process of ROI construction was semi-automatic: Each step was carefully checked by M.B. and potential errors introduced by the algorithm were manually corrected when possible. These corrections addressed issues such as mislabeling another sulcus as the STS, inclusion of regions beyond the horizontal segment or incorporation of parts of other sulci. F.L and C.M. double-checked the results. Participants were excluded when no agreement could be reached between raters or when STS segmentation was incorrect due to poor data quality. Ultimately, ROIs were successfully extracted in both hemispheres for 141 out of 159 neonates.

#### Time series cleaning

Despite the preprocessing done by the dHCP comprising motion correction and temporally denoising (Andersson et al. 2001, 2017, 2018; Fitzgibbon et al. 2020), some data might remain noisy due to residual artefacts (magnetic field perturbation secondary to head movements and physiological processes, such as heartbeat, breathing etc.). To avoid sudden jumps that destroy correlation, especially between antero-posterior regions due to the typical pitch movement observed in infants, we applied the same method as Eyre et al. (2021). Specifically, we retained a continuous sub-sample of 1,600 motion-corrected volumes in each subject (≈ 70% of the time series, ≈ 10 min of acquisition time). The time-series length was thus fixed and consistent across participants. To achieve this, we extracted 700 overlapping series of 1600 volumes (from the 2300 total volumes), each using a sliding window approach. The least noisy section was selected based on movement parameters associated with the images (three translation and three rotation parameters). For each series, we calculated the framewise displacement (FD(t) between volumes as the sum of the absolute values of the motion parameters and a threshold T was calculated for each series, following Eyre *et al’s.* method (2021), such as:

$$\:T=1.5\times\:IQR+C$$

where IQR represents the interquartile range, and C represents the value of the 75th centile.

Then, we calculated the number of points within each series with a FD higher than *T*. This corresponds to the number of outliers within the subsamples. Given that some series had the same number of outliers, we ranked them on the sum of the distances between the threshold and FD(t) and retained the series with the smallest combined distance between the threshold and FD(t). If more than 10% of the volumes (160 points) were outliers, the subject was excluded from the study. This method excluded 25 subjects. In the remaining 116 subjects, we checked the absence of correlation between gestational age at birth and the number of outliers detected in the time series (Pearson correlation, *p* = 0.3) and with the average framewise displacement of the retained time series (Pearson correlation, *p* = 0.17). Finally, we applied a band-pass filter (0.008–0.09 Hz) to each time series to reduce physiological noise (Yuen et al. 2019).

#### Connectivity analysis at the subject level in the native space

All connectivity and statistical analyses were conducted in each infant’s native space using the Python modules Nilearn (v.0.7.1) and Scipy (v.1.6.3). The time-series of all voxels in a ROI were averaged together to constitute the seed time-series. The seed-to-voxel correlation maps were computed across the whole brain using the Pearson coefficient (Mahadevan et al. 2020), and then z-scored. The mean time-series of the white matter mask was also computed and included in the model as a non-interest confound.

#### Normalization

All normalization steps were performed using SPM12 software (https://www.fil.ion.ucl.ac.uk/spm/). Once correlation maps had been calculated for each subject, all subjects were normalized to a reference space. We choose the infant template proposed by Kabdebon et al. (2014) because it has been parceled in anatomical regions following Tzourio-Mazoyer et al’s recommendations (2002) in the Automated Anatomical Labeling (AAL) atlas in adults and has been used in our other functional studies (Adibpour et al. 2020). To create a template reflecting our neonatal population, we first normalized toward Kabdebon et al’s template, 32 neonates, randomly selecting 8 neonates from each of 4 groups defined by their gestational age: < 32, [32, 35], [36, 38], >= 38. The obtained deformation field was applied to the grey matter, white matter and cerebro-spinal fluid distributed within dHCP (Draw-EM algorithm) and a mean image with these three compartments was obtained. All original T2w volumes were then normalized to the mean image with a quadrilinear interpolation. The same deformation field was applied to the ROI-brain correlation maps to obtain all maps in the same reference space.

#### Creation of a symmetrical template to study left–right functional asymmetries

In order to compare the connectivity from the left and right STS, the correlation maps had to be registered in a perfectly symmetrical space, i.e. free of any hemispheric differences (petalia, Yakovlean torque, etc…,Kabdebon et al. 2014). Thus, the infant template, described above was made symmetrical according to the procedure used by Didelot et al. (2010):

- The original template (orTemplate) was flipped along the x axis (fTemplate) and summed to the orTemplate. This creates a symmetrical image named orfTemplate.
- The orTemplate and fTemplate were coregistered on orfTemplate, then the mean of these two images was computed, creating the cTemplate.
- This last image was flipped again creating the fcTemplate. Finally, the mean of the cTemplate and fcTemplate was then computed to obtain the symmetrical template (sTemplate).

For each child, the following steps were then followed:

- The anatomical images were flipped along the x-axis.
- The original and flipped images were normalized to the template created above and a deformation field was obtained for each image, which was applied on the corresponding left-STS correlation maps and flipped right-STS correlation maps. This assures that both images were aligned and had both been transformed through a normalization process toward the symmetrical template. The normalized left STS correlation maps and flipped right STS correlation maps were then compared.

### Statistical analysis

**Fig. 2:**
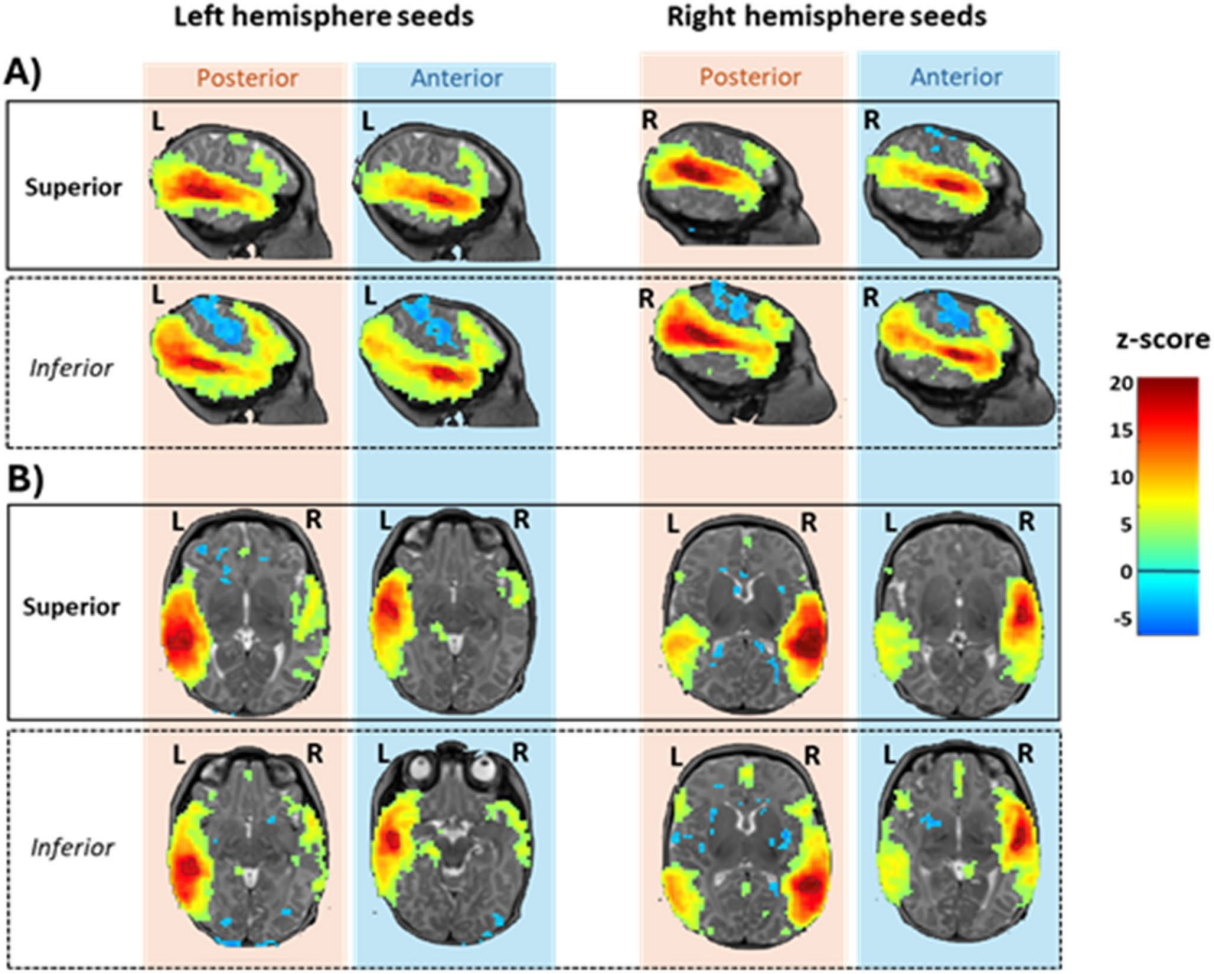
Functional connectivity of the left and right four ROIs. Clusters showing significant positive and negative correlations with the mean BOLD response of the ROIs (*p* < 0.001 at the peak, pFDR < 0.05 at the cluster level) are displayed on sagittal (**A**) and horizontal slices (**B**) of an individual neonate. The chosen slices correspond to the maximum correlation peak for each analysis, trivially within the ROI being analyzed. Significant clusters are observed in frontal areas, particularly for the inferior ROIs, while significant negative correlations are noted around the central sulcus for inferior seeds. On the horizontal slices (**B**) contra-lateral correlation appears stronger for right ROIs (last two columns) than for left ROIs (first two columns) but see statistical comparisons in Fig. 4. *L* Left, *R* Right

**Fig. 3:**
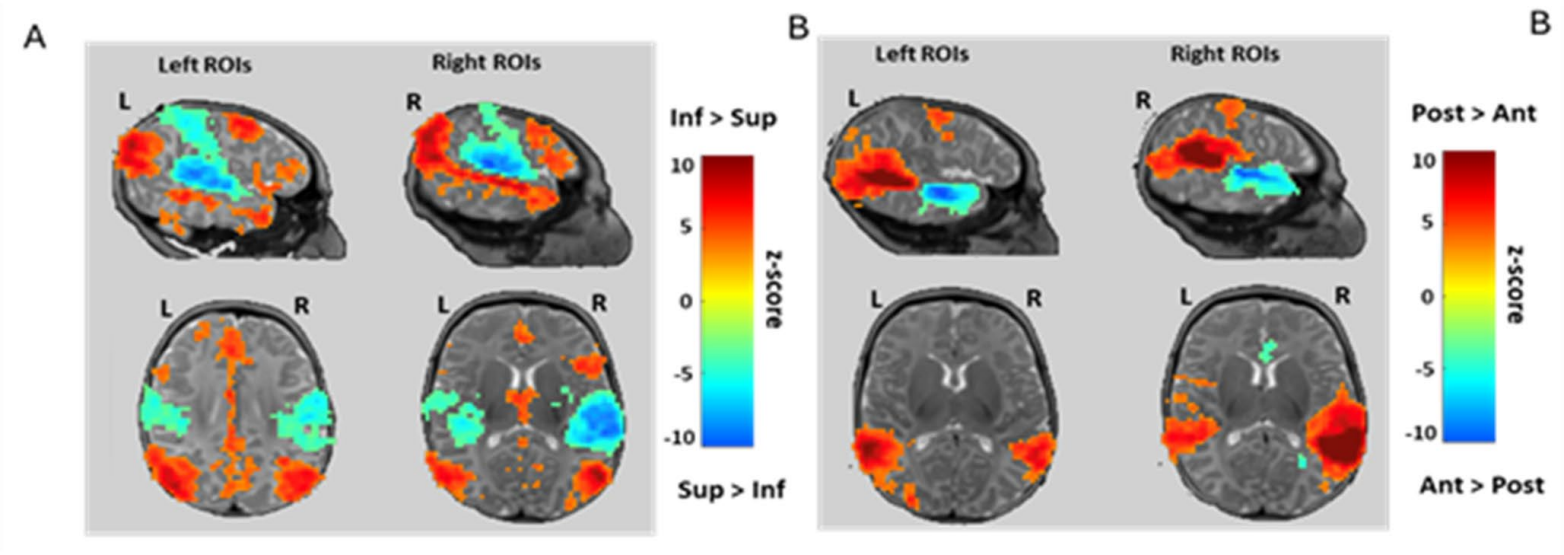
Differences in functional connectivity between superior and inferior ROIs (**A**) and between posterior and anterior ROIs (**B**) (*p* < 0.001 at the peak, pFDR < 0.05 at the cluster level). Each column represents separate analyses for the left and right ROIs. The slices were selected at the coordinates of the peak difference. In A, the inferior ROIs show significantly stronger correlations with inferior parietal regions and prefrontal regions and less with regions bordering the central sulcus than superior ROIs. In B, although the mean BOLD fluctuation in both anterior and posterior ROIs correlated with the contralateral STS as a whole (Fig. 2), the absence of blue clusters in the contralateral hemisphere indicates that posterior ROIs have stronger interhemispheric connectivity than anterior ROIs

**Fig. 4:**
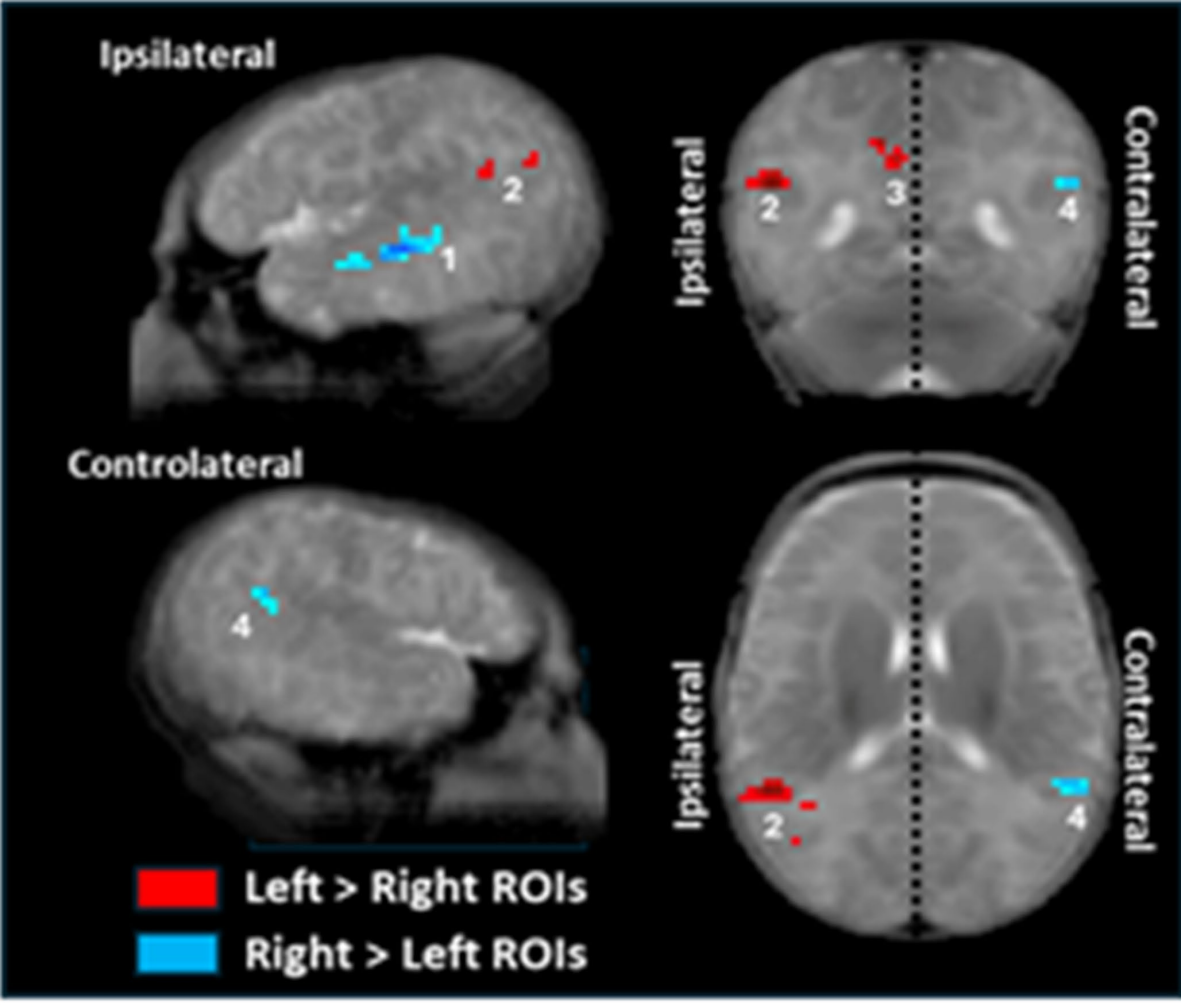
Differences in functional connectivity between left and right STS (*p* < 0.001 at the peak, pFDR < 0.05 at the cluster level). To allow direct comparison, both left and right seeds were mapped onto the same hemisphere of a symmetrized brain template. In the displayed images, the left side of the brain corresponds to ipsilateral connectivity (i.e., same hemisphere as the ROI), and the right side corresponds to contralateral connectivity. Therefore, the left–right orientation in the figure does not correspond to the actual hemispheric anatomy. Blue clusters indicate regions more strongly connected to the right STS compared to the left, and red clusters reflect the opposite comparison. The right STS showed stronger local connectivity in its medial portion (1), while the left STS was more connected with its posterior vicinity (2) and the precuneus (3). Additionally, the right STS exhibited stronger connectivity than the left with its contralateral posterior region (4)

All correlation maps in the template space were smoothed using a $\:3\times\:3\times\:3mm$ Gaussian kernel, then entered in second-level group ANOVAs using SPM12. First, whole-brain functional connectivity analyses were performed separately for each seed, yielding the connectivity maps shown in Fig. 2. Next, to isolate ROI specific connectivity, we compared connectivity maps in pairs in each hemisphere: superior vs. inferior ROIs to distinguish auditory and linguistic networks (Fig. 3A), and anterior vs. posterior ROIs to understand the development of the STAP (Fig. 3B). We also examined hemispheric differences by comparing the left and right STS, using the entire sulcus as the region of interest (Fig. 4). For this analysis, functional connectivity maps from the left STS were compared with those from the right STS, after flipping the right hemisphere maps along the midline. To account for anatomical asymmetries, both hemispheres were first normalized to a symmetrical template to ensure accurate alignment as explained above.

**Fig. 5:**
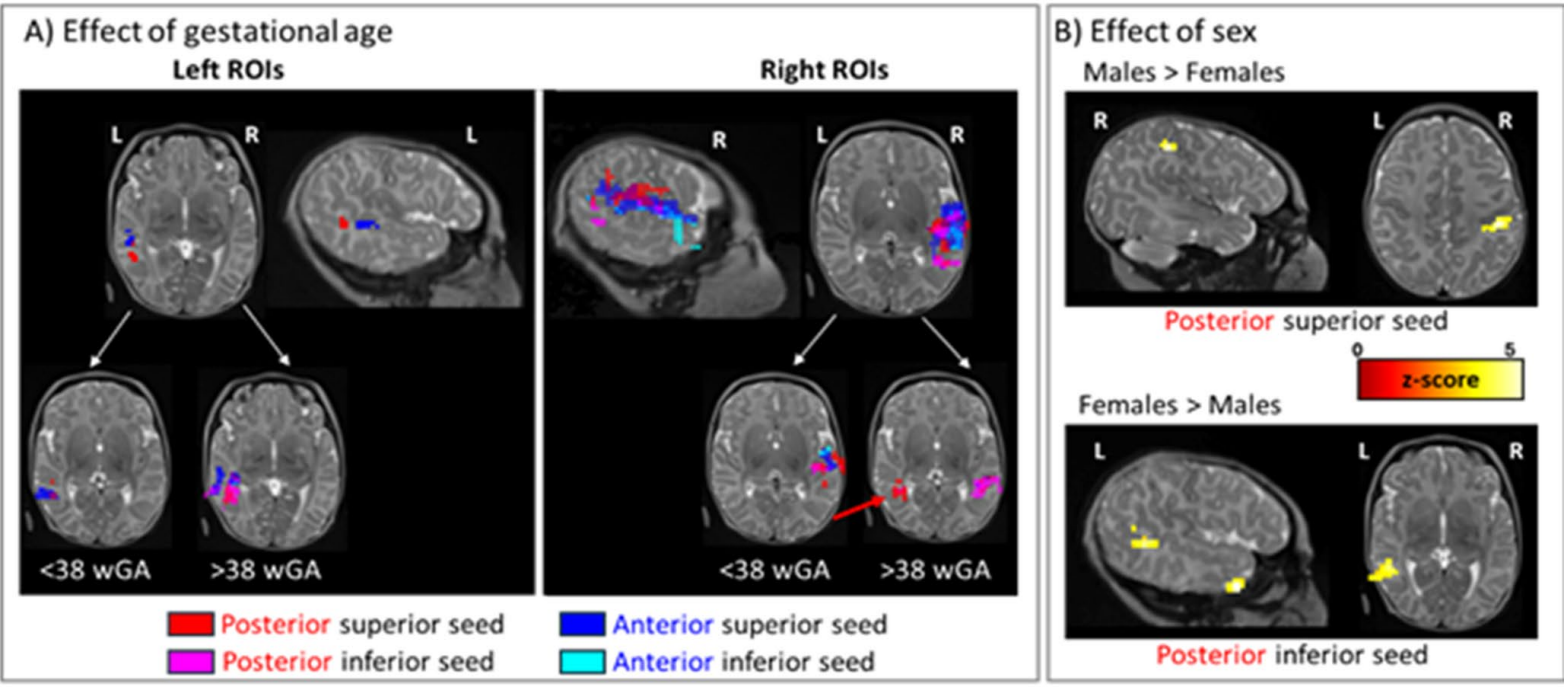
**A** Positive effect of longer gestation on the functional connectivity at term. Each color represents the significant clusters (*p* < 0.001 at the peak level and pFDR < 0.05 at the cluster level) identified in the analyses for each ROI and each hemisphere projected onto an individual newborn brain. Gestational age has a modest effect on functional connectivity for left ROIS contrasting with the sizeable effect for right ROIs whether the analysis concerns the whole group (top row) or each subgroup (bottom row). In addition, neonates in the term range displayed a stronger connectivity with a longer gestation of the right posterior ROIs with the contralateral left region (red arrow). **B** Effect of sex on the functional connectivity of each ROI. Male neonates show stronger connectivity from the right posterior superior ROI to the ipsilateral postcentral gyrus (top row) whereas females show a higher correlation between the left posterior inferior ROI with its inferior vicinity and the temporal pole (bottom row)

Finally, we assessed the effects of gestational age at birth and sex on functional connectivity using separate regression analyses for each ROI, with both variables entered as regressors allowing us to examine the effect of one factor while controlling for the other (Fig. 5). To determine whether the observed effects of gestational age were limited to a specific age window, we repeated these analyses within two subgroups, using 38 wGA as the cutoff. This threshold was chosen because neonates born between 37 and 38 wGA, classified as early-term, are still at a higher risk of developmental delays, particularly in communication, compared to their later full-term peers (Hochstedler et al. 2021; Sengupta et al. 2013; Vohr 2013). Since our two factors appeared to differentially impact right and left STS connectivity, we performed a follow-up analysis to directly assess hemispheric differences. Specifically, we conducted a regression analysis on maps representing the difference in functional connectivity between the left STS and the flipped right STS, using the entire sulcus as the region of interest and including both variables as regressors as above.

Results were reported when voxels were significant at *p* < 0.001 and formed a contiguous cluster whose extent was significant at *p* < 0.05, FDR corrected for multiple comparisons across the entire brain volume. To present the findings in a comprehensive and interpretable way, we applied two complementary strategies depending on the size and extent of the significant clusters. Most comparisons yielded large clusters, which are displayed in the corresponding figures. For smaller clusters, we report their size and statistical values directly in the text.

## Results

### ROI-to-voxel analysis

As expected given previous publications (Eyre et al. 2021; Perani et al. 2011), each ROI showed a significant correlation with the contralateral region (Fig. 2B). This correlation was not limited to the exact contralateral region but extended to the entire STS on the other side. Moreover, inferior ROIs (both left and right) displayed significant positive correlations with the BOLD signal in the inferior parietal and prefrontal areas and negative correlations with the region bordering the central sulcus. Right ROIs showed a strong correlation with the contralateral left regions, while the left ROIs appeared mostly correlated with ipsilateral regions.

### Comparisons between rois

#### Inferior vs. superior ROI (significant clusters are presented in Fig. 3A)

Trivially, each seed exhibited stronger correlations within itself (maxima of the blue and right clusters along the STS). Each ROI also showed a stronger correlation with its specific contralateral region. But the most striking difference in the ROIs connectivity appeared in the more distant correlations: the inferior temporal ROIs were significantly less correlated with the pre- and postcentral gyri bilaterally (blue clusters in Fig. 3A) than the superior ROIs, but more so with several regions in the inferior parietal and frontal lobes, along the dorsal peri-sylvian pathway (red clusters in Fig. 3).

#### Anterior vs. posterior rois (Fig. 3B)

In Fig. 3B, red regions indicate areas more strongly correlated with the posterior STS seeds than with the anterior seeds, while blue regions indicate the reverse comparison. Strikingly, red clusters—but not blue clusters—are observed contralaterally. This suggests that the posterior STS seeds were more strongly correlated with contralateral regions, particularly posterior temporal areas, whereas anterior and posterior ROIs showed similar correlations with contralateral anterior regions.

In other words, the mean BOLD signal in the posterior ROIs was more in phase with activity in the contralateral temporal lobe than was the case for the anterior ROIs. Additionally, the posterior ROIs exhibited stronger connectivity with the precentral gyrus compared to the anterior ROIs. This pattern was consistent for both left and right hemisphere seeds.

#### Left vs right rois

To perform this comparison, connectivity maps from left and right STS ROIs were projected onto the same hemisphere using a symmetrical brain template (see method). As a result in Fig. 4, the left side represents ipsilateral connectivity (within the same hemisphere as the seed), and the right side represents contralateral connectivity—regardless of the actual hemispheric origin of the seed.

We observed distinct differences in functional connectivity between the left and right STS (see the labeled clusters (1)–(4) in Fig. 4). The right STS was more strongly connected with: the adjacent middle temporal gyrus (43 voxels, pFDR = 0.001, z = 4.95, blue cluster (1) in Fig. 4) and a contralateral posterior region in the left hemisphere (24 voxels, pFDR = 0.029, z = 4.62, blue cluster (4)). Conversely, the left STS showed stronger connectivity extending posteriorly to the posterior part of the left STS (34 voxels, pFDR = 0.002, z = 4.50) and left angular gyrus (27 voxels, pFDR = 0.006, z = 4.80, red cluster (2)), as well to the precuneus (47 voxels, pFDR < 0.001, z = 4.48, red cluster (3)).

### Effect of gestational age and sex

After characterizing the functional connectivity of our ROIs, we examined whether sex (Fig. 5A) and gestational age at birth (Fig. 5B) influenced STS connectivity. To do so, we conducted linear regression analyses using both variables as regressors.

#### Gestational age

All significant effects were positive, i.e. longer gestation was associated with stronger functional connectivity. Considering the left ROIs, the effect of longer gestational age was modest, confined to the connectivity of the superior ROIs (Fig. 5A, top left and Table 2 left columns). Dividing the group at 38 wGA to assess the effect of reaching full-term yielded similar results in both subgroups (Fig. 5A, bottom left).

For right hemisphere ROIs, a large linear effect of gestational age was observed with stronger local correlations in the right temporal region for infants born at a later gestational age (Fig. 5A top right and Table 2 right columns). This pattern was consistent across both subgroups (Fig. 5A, bottom right). In addition to these shared effects, we identified additional effects within the full-term group (i.e. [38–41.7] wGA). Specifically, longer gestation was associated with increased connectivity between the right posterior inferior STS ROI and the ipsilateral temporal pole (59 voxels, pFDR < 0.001, z = 5.31). Furthermore, for both right posterior ROIs, we observed a linear increase in inter-hemispheric connectivity with the left contralateral posterior region (inferior ROI: 39 voxels, pFDR = 0.002, z = 4.36 and superior ROI: 72 voxels, pFDR < 0.001, z = 4.52, red cluster in Fig. 5A).

**Table 2:** Significant clusters showing a significant positive gestational age effect across the entire range [24.3–41.7] wGA for each seed

	Left ROIs			Right Rois		
Seed	Voxel Count	pFDR	z-score at peak	Voxel Count	pFDR	z-score at peak
Anterior Inferior				200	< 0.001	5.38
				27	0.016	4.47
				37	0.005	4.13
Anterior Superior	26	0.024	3.88	621	< 0.001	6.47
Posterior Inferior				83	< 0.001	4.96
				98	< 0.001	4.78
Posterior Superior	37	0.005	3.82	487	< 0.001	5.13

All clusters were located in the temporal region and were ipsilateral to the respective seeds. No significant negative clusters were observed

#### Sex (Fig. 5B)

This factor mainly modulated the functional connectivity of the posterior ROIs. Notably female neonates displayed stronger local functional connectivity than males in the linguistic bank of the STS: the left posterior inferior ROI was more correlated to its above and below vicinity (98 voxels, pFDR < 0.001, z = 4.68 and 45 voxels, pFDR = 0.001, z = 4.47) as well as both inferior ROIs to the temporal pole (36 voxels, pFDR < 0.002, z = 5.24 for the left posterior ROI and 31 voxels, pFDR = 0.013, z = 4.66 for the left anterior ROI). Male neonates had stronger correlations than females between the right posterior superior ROI and the postcentral region (32 voxels, pFDR = 0.012, z = 4.85).

The same pattern in favor of female neonates was observed when considering only the full-term subgroup: the left inferior posterior ROI was more connected to its posterior vicinity (210 voxels, pFDR < 0.001, z = 4.85), and both inferior ROIs to the temporal pole (posterior ROI: 39 voxels, pFDR = 0.004, z = 4.63, anterior ROI: 38 voxels, pFDR = 0.006, z = 5.31). In the right hemisphere, sex, favoring females, also modulated the connectivity of the inferior ROIs: the anterior ROI with the temporal pole (34 voxels, pFDR = 0.013, z = 4.44) and the posterior ROI to the gyrus medial part (56 voxels, pFDR = 0.001, z = 5.22) and to the right hippocampus (30 voxels, pFDR = 0.014, z = 4.71). There was no significant cluster in the reverse direction.

By contrast in neonates born before 38 wGA, there was no stronger functional connectivity for female relative to male neonates. The only significant difference was in the reverse direction: the right superior posterior ROI was more connected to the right postcentral region (41 voxels, pFDR = 0.002, z = 4.40) in male than in female preterm neonates.

#### Effect of gestational age and sex on hemispheric asymmetries

Since our two factors appeared to have a different impact on right and left STS connectivity, we performed a follow-up analysis to assess whether hemispheric differences were significant. Gestational age did not significantly affected more one hemisphere than the other. However, we observed a slight advantage for female infants, who showed stronger connectivity between the right STS and the contralateral anterior insula (20 voxels, pFDR = 0.04, z = 4.38).

## Discussion

**Fig. 6:**
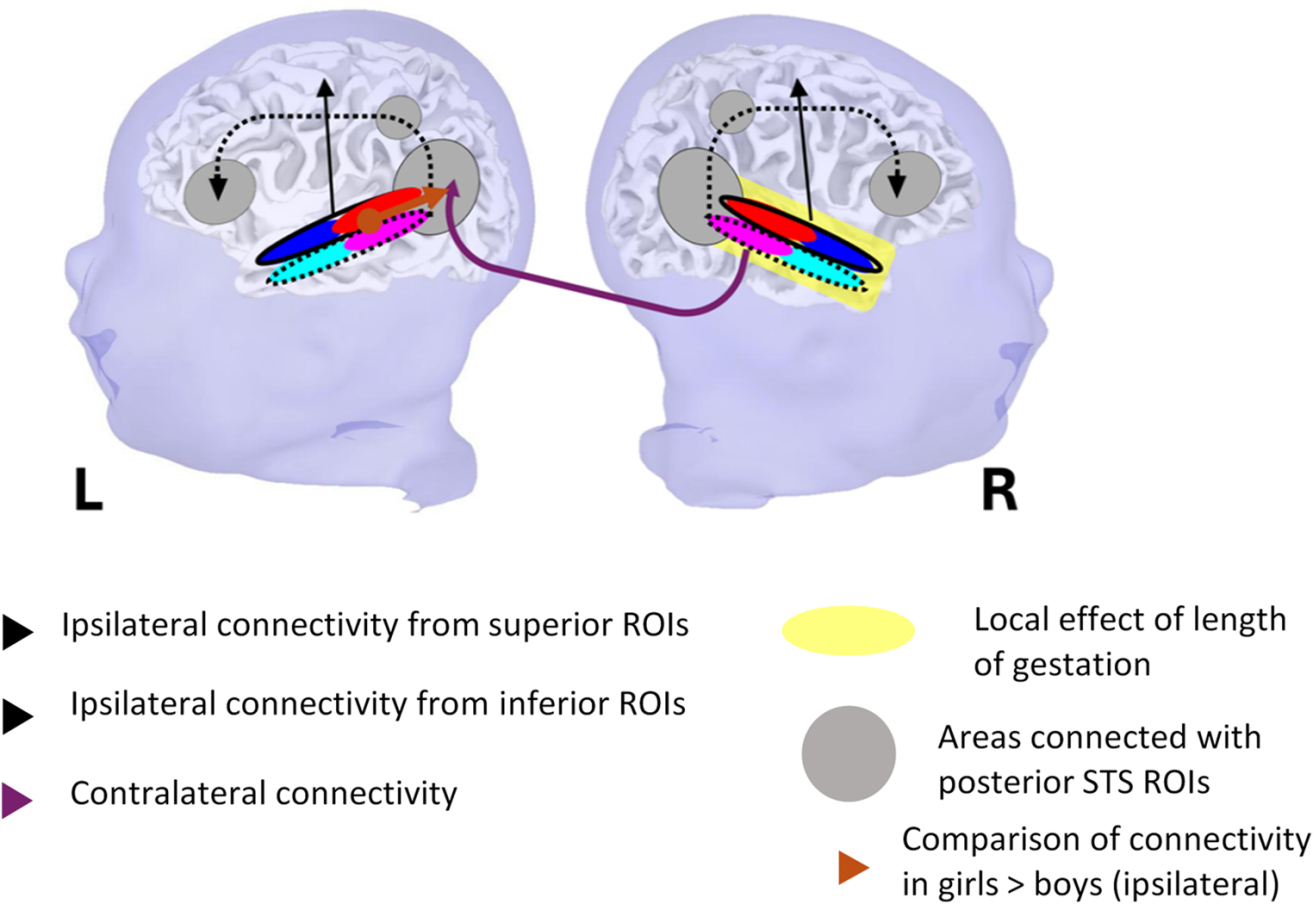
Schematic view of the functional connectivity of the STS in neonates. This functional architecture was roughly similar in both hemispheres except that the right STS was more connected to the left posterior region than the left to the right, a feature that was notably observed with longer gestation in the term range. The left posterior temporal region thus appeared to be an important functional hub, whose connectivity is also affected by sex (stronger connectivity in female than male neonates). Eventually, the length of gestation had a significant and progressive effect on the local connectivity across the right superior temporal region (shown in yellow). Dotted lines indicate the rois and shematic connectivity from ventral (inferior) STS rois; plain lines dorsal (superior) rois.

Given the STS’s central role in human communication, we aimed to characterize the auditory and linguistic networks at term-equivalent age, a critical milestone in brain maturation. To do so, we analyzed STS connectivity using ROIs tailored to the specific STS morphology of each neonate. This individualized approach allowed us to compute connectivity in each infant’s native space while accounting for STS subregional organization. This ensured greater anatomical precision and facilitated meaningful comparisons between resting-state connectivity patterns and previous findings from task-based studies. By comparing neonates born at different gestational ages—but all scanned at term-equivalent age—we were also able to assess the potential impact of early auditory experience on the development of this region. Figure 6 provides a visual summary of our main findings, which we outline below prior to discussing their broader implications.

1. There was a striking difference in the functional connectivity between the two banks of the STS (Fig. 3A). The inferior bank was connected to the inferior parietal and frontal areas, which, in the left hemisphere, are part of the classical linguistic network. By contrast, the BOLD modulations of the superior bank appeared more correlated with the areas bordering the central sulcus indicating a clear functional division between the two STS banks.

2) The posterior superior temporal region was connected with a range of both nearby and distant brain areas (Fig. 3). Notably, as illustrated in Fig. 4, the left posterior temporal region exhibited functional connectivity with the ipsilateral STS ROI (cluster 2 and 3) as well as the contralateral right STS ROI (cluster 4), suggesting its role as a functional hub (Figs. 3B and 4).
3) Gestational length significantly influenced functional homogeneity within the temporal lobe, particularly in the right hemisphere, i.e. longer gestation was associated with a greater extent of temporal regions exhibiting BOLD signal correlations with the STS banks. This gestational age effect remained significant even when analyses were restricted to the full-term range. During this period, we also observed an increase in contralateral connectivity in addition to the enhanced local correlations (Fig. 5A, bottom right).
4) Female neonates exhibited more widespread connectivity from the left inferior STS ROIs—corresponding to the linguistic bank—toward other brain areas.

Although attributing functions to connectivity patterns in immature infants remains challenging, these results provide valuable insights. Considered alongside previous findings in infants and adults, they help inform hypotheses about how auditory and linguistic networks develop, as discussed below.

### The linguistic network at term-equivalent age

Our understanding of how infant brain circuitry supports language acquisition remains limited. Based on our STS connectivity analyses, we would like to highlight three findings that may contribute to this question. While these observations likely require further investigation, they align with task-based studies and offer new perspectives on the early organization of the linguistic network.

#### STS connectivity with frontal areas

The clear connectivity of the inferior bank of the STS with the frontal regions confirms the early establishment of adult-like networks encompassing distant high-level regions. Long-range connectivity with frontal areas has also been reported for other networks both anatomically (Vasung et al. 2019) and functionally in resting-state studies (Barttfeld et al. 2018; Doria et al. 2010; Fransson et al. 2007, 2011; Kamps et al. 2020; Li et al. 2020; Sours et al. 2017; Sylvester et al. 2023). These findings are also consistent with task-based activation studies: in 3-month-old infants and full-term neonates using fMRI (Dehaene-Lambertz et al. 2002; Perani et al. 2010, 2011) and even before term, at 29–30 wGA, using Near InfraRed Spectroscopy (NIRS) (Mahmoudzadeh et al. 2013), frontal activations have been observed in response to speech stimuli. Altogether, this growing body of evidence indicates that the frontal cortex, although immature, is functional and integrated into broader cortical networks from very early in development (Mahmoudzadeh et al. 2013; Sylvester et al. 2023) and challenge earlier studies, such as Perani et al. (2011) which reported inter-hemispheric connectivity between homologous areas but found no anterior-posterior correlations. These discrepancies are likely attributable to small sample sizes and the technical limitations of early infant neuroimaging. In contrast, advances in MRI acquisition, scanner technology, and analytical pipelines—particularly those developed within the dHCP—now enable more reliable detection of temporo-frontal connectivity in neonates.

Due to the challenges in tracking the frontal end of the arcuate fasciculus—particularly because of its intersection with the more mature corticospinal tract (Dubois et al. 2016)—some authors have downplayed the involvement of the dorsal pathway in pre-verbal infants (Brauer et al. 2013; Perani et al. 2011). Although our resting-state analyses cannot isolate the specific involvement of each pathway, the posterior STS ROIs exhibited extended connectivity with parietal and frontal regions typically associated with the dorsal stream. This suggests that the core architecture of the language network is already established at birth, with later developmental changes reflecting maturational refinement rather than structural reorganization.

#### left–right asymmetries in the posterior temporal region?

As language is generally described as relying mostly on the left hemisphere, we looked for hemispheric asymmetries and created a symmetrical template to perform left–right comparisons while controlling for spurious differences due to the Yakovlean torque that pushes up and forward the right hemisphere relative to the left (Yakovlev 1962). Our results showed mostly comparable connectivity patterns in both hemispheres. This is consistent with our own previous studies using speech stimuli in infants (Dehaene-Lambertz et al. 2002, 2006, 2010; Mahmoudzadeh et al. 2013), where we mainly observed bilateral activations, with even larger responses to speech syllables in most of the right hemisphere in preterm neonates. These findings were also reported in Heschl’s gyrus in full-term neonates (Perani et al. 2011). Although hemispheric asymmetry in the language network undoubtedly increases with age (Emerson et al. 2016; Shultz et al. 2014), we have consistently observed a left-hemispheric bias in the posterior temporal region in our previous activation studies (Dehaene-Lambertz et al. 2010). Here, the left posterior temporal region also appeared to occupy a peculiar position as a functional hub connected to numerous regions.

The left posterior superior temporal region displayed stronger ipsilateral connectivity than the right, with its posterior and inferior vicinity, the superior part of the precentral region, and the precuneus (Figs. 3B and 4). Interestingly, female sex and longer gestation were associated with higher connectivity of the temporal ROIs with the left posterior STS and middle temporal gyrus (Fig. 5). Finally, a longer gestation also accentuated the correlation of the right STS with the left posterior region suggesting an asymmetric inter-hemispheric transfer from the right to the left hemisphere (Fig. 5A bottom row). The lack of significant difference when directly comparing left and right STS connectivity might be related to a lack of sensitivity for this second-order comparison.

Such asymmetries may reflect structural biases in inter-hemispheric transfer, particularly favoring communication from the right to the left auditory cortex through the corpus callosum as reported in infants (Adibpour et al. 2020) and adults (Andoh et al. 2015; Gotts et al. 2013; Karpychev et al. 2022). In adults, repetitive stimulation of the right auditory cortex with TMS modulates its connectivity with the left contralateral temporal cortex but not the reverse. It has been explained by asymmetric callosal fibers favoring transfer toward the left temporal region (Andoh et al. 2015). Karpychev et al. (2022) recently brought a new argument to the role of the corpus callosum in language lateralization, showing the relation between language lateralization in adults and the volume of the temporal callosal fibers in the splenium measured with constrained spherical deconvolution (CSD). As the left posterior temporal region is notably involved in phonetic coding and intelligibility (Hickok and Poeppel 2007; Vagharchakian et al. 2012), these results underline the centrality of this region in auditory-linguistic networks from the outset. The early and important role of the callosal inter-hemispheric transfer is also supported by the correlation between FA changes in splenium and linguistic performances in toddlers (Swanson et al. 2017). Children with greater acceleration in FA changes between 6 and 24 months in the posterior part of the corpus callosum, but not in classical language tracts, were greater talkers at 24 months. Finally, the abnormalities often detected in the posterior corpus callosum in preterm adolescents with language difficulties (Northam et al. 2012) might also be linked to the corpus callosum’s role in connecting the right temporal regions to the left posterior temporal hub. Further studies, notably using the functional and anatomical images of the dHCP dataset, might clarify the role of the corpus callosum.

Our analysis also revealed a correlation of the left posterior temporal region with the precuneus. The precuneus, a densely connected region is involved in many high-level tasks in adults, notably related to self- generated representations during rest. Therefore, its role might have been assumed to be negligible in infants. However, its functional connectivity already demonstrates in neonates a similar specificity to what is observed in adults (Sylvester et al. 2023), and it has been shown to activate in 3-month-old infants listening to their native language compared to backward speech (Dehaene-Lambertz et al. 2002). In adults, it is observed in the retrieval of items learnt during infancy, in association with the left temporal operculum (Fiebach et al. 2003), suggesting an early functional role of the precuneus that needs to be further explored during infancy.

#### Auditory-sensory-motor connectivity

The sensory-motor regions bordering the central sulcus were observed in several of our connectivity comparisons with significantly stronger coupling observed for the superior and posterior ROIs compared to the inferior and anterior ones (Fig. 3A–B). Although these effects are relative (i.e. observed through comparisons between ROIs), they are consistent with behavioral and EEG studies suggesting certain mapping between auditory, visual and motor speech representations in infants (Bristow et al. 2008; Choi et al. 2019; Patterson and Werker 2003). For example, holding a pacifier that blocks specific tongue movements modulates syllable perception in infants (Choi et al. 2019, 2021). The early coupling between speech perception and production systems warrants further investigation, particularly in clinical contexts where mechanical interventions—such as feeding tubes or intubation —may restrict orofacial movements during a critical period for sensorimotor integration.

### Effect of gestational age on the STS functional connectivity

Beyond the increased connectivity with the left posterior temporal region reported above, gestational age had a linear impact on the functional connectivity of the STS: Longer pregnancies were associated with larger clusters in the temporal lobe exhibiting BOLD fluctuations correlated with the right STS. This effect remained observable even within the restricted term range (38–41.7 weeks of gestational age in our sample).

Although the effect was observed primarily in the right STS (Fig. 4), no significant difference emerged when directly comparing left and right STS connectivity suggesting that the left temporal lobe may follow a similar developmental trajectory. Nevertheless, the absence of a statistically significant hemispheric difference may reflect limited statistical power, as a right-dominant effect would be consistent with previous evidence indicating faster maturation of the right hemisphere: Most sulci, including the STS, appear earlier in the right hemisphere (Chi et al. 1977; de Vareilles et al. 2023; Habas et al. 2012); cerebral blood flow, as measured by single photon emission computed tomography (SPECT) is higher in the right hemisphere until around three years of age (Chiron 1997); maturation indices derived from T1- and T2-weighted MRI and diffusion tensor imaging reveal more advanced development in structures such as the right Heschl’s gyrus and *planum temporale* (Leroy et al. 2011). This fast development, notably during the age range considered here, may partly explain the greater sensitivity of the right STS to environmental influences.

This environmental effect on right STS connectivity may be linked to the specific characteristics of the auditory environment in utero, where the abdominal wall acts as a low-pass filter for external sounds (Querleu et al. 1988). As a result, slow prosodic information becomes the dominant component of speech perception in the womb, potentially favoring processing in the right auditory regions, which have been shown to be more sensitive to low temporal modulation frequencies (Boemio et al. 2005; Zatorre et al. 2004). This would explain the linear relationship observed between gestational length and the right superior temporal connectivity.

### Effect of sex on the posterior STS functional connectivity

The effect of sex on brain development, and notably on language, has been widely discussed, often with mixed results (Christians et al. 2023; Etchell et al. 2018; Hirnstein et al. 2019). First, anatomical differences between male and female brains have been observed, even when controlling for overall brain and body size (Williams et al. 2021b). Second, girls tend to exhibit better language and social abilities on average (Frank et al. 2017) and are less frequently diagnosed with oral and written language disorders Third, in the context of prematurity, girls are classically considered less vulnerable to negative developmental consequences (Etchell et al. 2018; Hirnstein et al. 2019) although this view has been challenged by a recent meta-analysis (Christians et al. 2023) Although it remains difficult to disentangle cultural from biological influences, several studies have reported early sex-related differences in brain maturation and stress reactivity (Bale 2016).

In our study, the effect of sex on STS functional connectivity was moderate. It was primarily characterized by stronger local connectivity, in female neonates compared to males, between the posterior inferior ROI and adjacent regions along the inferior bank of the STS—areas commonly associated with early linguistic processing. Given the critical role of these regions in speech processing, this enhanced functional connectivity in females may contribute to their greater resilience to language-related difficulties.

### Limitations and conclusion

The present study relied on a semi-automatic delineation of STS morphology in each individual enabling us to investigate specific networks while accounting for intersubject anatomical variability. This individualized approach allows for the extraction of targeted circuits that can be meaningfully related to task-based findings. However, ROI volumes naturally vary across individuals and even within individuals (e.g., anterior vs. posterior regions, depending on the STAP boundary location, or left vs. right due to the typically larger right STS). Such variations may influence sensitivity: larger ROIs might be less functionally homogeneous, while also potentially providing more robust signal estimates. Despite the relatively large sample size (116 infants), some second-order comparisons may still lack sufficient statistical power. Future studies leveraging larger cohorts and more fully automated processing pipelines will be instrumental in confirming—or refining—the hypotheses proposed in this work.

We found that the main divisions of the peri-sylvian networks (inferior vs. superior, anterior vs. posterior, and left vs. right) were already evident at term-equivalent age and remained remarkably stable despite significant environmental differences. The exception lies in the right temporal lobe, which showed greater sensitivity to gestational age. These findings underscore the importance of studying early development to the disentangle environmental and intrinsic contributions to the establishment of these networks.

## Data Availability

Data were provided by the developing Human Connectome Project, KCL-Imperial-Oxford Consortium funded by the European Research Council under the European Union Seventh Framework Programme (FP/2007-2013)/ERC Grant Agreement no. [319456]
